# Real‐world sequential treatment patterns and clinical outcomes among patients with advanced urothelial carcinoma in Japan

**DOI:** 10.1111/iju.15411

**Published:** 2024-02-01

**Authors:** Yuki Kita, Hikari Otsuka, Katsuhiro Ito, Takuto Hara, Soichiro Shimura, Takashi Kawahara, Minoru Kato, Sojun Kanamaru, Koji Inoue, Hiroki Ito, Atsushi Igarashi, Tomokazu Sazuka, Dai Takamatsu, Kohei Hashimoto, Takashige Abe, Sei Naito, Yoshiyuki Matsui, Hiroyuki Nishiyama, Hiroshi Kitamura, Takashi Kobayashi, the Japan Urological Oncology Group

**Affiliations:** ^1^ Department of Urology Kyoto University Graduate School of Medicine Kyoto Japan; ^2^ Department of Urology, Tazuke Kofukai Medical Research Institute Kitano Hospital Osaka Japan; ^3^ Department of Urology Kobe University Kobe Japan; ^4^ Department of Urology Kitasato University Tokyo Japan; ^5^ Department of Urology University of Tsukuba Tsukuba Japan; ^6^ Department of Urology Osaka Metropolitan University Osaka Japan; ^7^ Department of Urology Kobe City Nishi‐Kobe Medical Center Kobe Japan; ^8^ Department of Urology Kurashiki Central Hospital Kurashiki Japan; ^9^ Department of Urology Yokohama City University Yokohama Japan; ^10^ Department of Urology Kobe City Medical Center General Hospital Kobe Japan; ^11^ Department of Urology Chiba University Chiba Japan; ^12^ Department of Urology Kyushu University Fukuoka Japan; ^13^ Department of Urology Sapporo Medical University Sapporo Japan; ^14^ Department of Urology Hokkaido University Sapporo Japan; ^15^ Department of Urology Yamagata University Tsuruoka Japan; ^16^ Department of Urology National Cancer Center Hospital Tokyo Japan; ^17^ Department of Urology University of Toyama Toyama Japan

**Keywords:** chemotherapy, enfortumab vedotin, immune checkpoint inhibitor, sequential treatment, urothelial carcinoma

## Abstract

**Objectives:**

Immune checkpoint inhibitors and enfortumab vedotin have opened new avenues for sequential treatment strategies for locally advanced/metastatic urothelial carcinoma (la/mUC). In the pre‐enfortumab vedotin era, many patients could not receive third‐line treatment owing to rapid disease progression and poor general status. This study aimed to analyze real‐world sequential treatment practices for la/mUC in Japan, with a focus on patients who do not receive third‐line treatment.

**Methods:**

We analyzed data for 1023 la/mUC patients diagnosed between January 2020 and December 2021 at 54 institutions from a Japanese nationwide cohort.

**Results:**

At the median follow‐up of 28.5 months, the median overall survival from first‐line initiation for 905 patients who received systemic anticancer treatment was 19.1 months. Among them, 81% and 32% received second‐ and third‐line treatment. Notably, 52% had their treatment terminated before the opportunity for third‐line treatment. Multivariate logistic regression analysis revealed that low performance status (≥1), elevated neutrophil‐to‐lymphocyte ratio (≥3), and low body mass index (<21 kg/m^2^) at the start of first‐line treatment were independent risk factors for not proceeding to third‐line treatment (*p* = 0.0024, 0.0069, and 0.0058, respectively). In this cohort, 33% had one of these factors, 36% had two, and 15% had all three.

**Conclusions:**

This study highlights the high frequency of factors associated with poor tolerance to anticancer treatment in la/mUC patients. The findings suggest the need to establish optimal sequential treatment strategies, maximizing efficacy within time and tolerance constraints, while concurrently providing strong supportive care, considering immunological and nutritional aspects.

**Abbreviations & Acronyms**
BMI: body mass index
BSC: best supportive care
CCr: creatinine clearance
CI: confidence interval
EV: enfortumab vedotin
la/m UC: locally advanced/metastatic urothelial carcinoma
NLR: neutrophil‐to‐lymphocyte ratio
PS: performance status

## INTRODUCTION

With immune checkpoint inhibitors^1^, ^2^ and antibody‐drug conjugates, such as enfortumab vedotin (EV),^3^ now clinically available, the treatment of locally advanced and metastatic urothelial carcinoma (la/mUC) has entered a new era of sequential treatment. However, recent real‐world studies of patients with la/mUC from the USA and The Netherlands have shown significant underutilization of systemic treatment and a high attrition rate from the first to subsequent lines of systemic therapy.^4^, ^5^, ^6^, ^7^ In multiple large cohort studies, almost half of the patients received no treatment for metastatic disease, while only approximately 15%–20% received second‐line therapy. Possible reasons for the lack of optimal receipt of systemic therapies and the high attrition rate include relatively toxic and moderately effective platinum‐based chemotherapy regimens, concerns about performance status (PS) and comorbidities in this patient population, communication barriers, lack of social support, and access to affordable healthcare.^8^

In Japan, where universal health insurance is provided, attrition for economic reasons or barriers to access to medical care is unlikely. An analysis of a Japanese cohort facilitates the identification of medical problems that influence the feasibility of la/mUC treatment. Thus, this unique situation makes it possible to evaluate the feasibility of la/mUC sequential treatment. In this study, we clarified the real‐world practice of sequential treatment of la/mUC in Japan while concurrently evaluating the clinical characteristics of patients who died before the opportunity for third‐line treatment.

## METHODS

### Study design

The clinical data were collected from the Japan Urological Oncology Group nationwide cohort of la/mUC patients. Briefly, this cohort comprised 1023 patients who were diagnosed as having la/mUC from January 2020 to December 2021 at 54 participating institutions. This dataset included information on the patients' clinical backgrounds, sequential treatment details, laboratory data, and treatment outcomes; survival data were collected in May 2023. There were no restrictions on the choice of treatment, which was in accordance with the policies at each institution. The metachronous metastasis group was defined as patients who had recurrence or metastasis after total cystectomy or nephroureterectomy, while the synchronous metastasis group was defined as patients with unresectable or metastatic urothelial carcinoma at diagnosis. This study was approved by the Institutional Review Board at Kyoto University Graduate School of Medicine (approval number R3245) and by the local Institutional Review Board at each participating institute. This study conformed to the provisions of the Declaration of Helsinki.

In Japan, pembrolizumab was approved for patients with failed platinum‐based chemotherapy in December 2017, avelumab was approved as maintenance treatment for patients with stable disease or better response to first‐line chemotherapy in February 2021, and EV was approved for patients with disease progression after platinum‐based chemotherapy and immune checkpoint inhibitors in November 2021. Sacituzumab govitecan and erdafitinib are not currently approved in Japan. Strictly speaking, switch maintenance immunotherapy with avelumab is considered as a part of first‐line treatment. In this study, however, avelumab was classified as second‐line therapy. This allowed avelumab treatment to be contrasted with second‐line pembrolizumab treatment as a post‐chemotherapy immunotherapy. Furthermore, EV after avelumab switch maintenance treatment can now be considered as third‐line therapy equivalent to EV after second‐line pembrolizumab treatment. For similar reasons, perioperative chemotherapy was classified as first‐line treatment if the patient received pembrolizumab for recurrence and metastasis within 1 year after radical surgery with perioperative chemotherapy.

The parameters evaluated in this study comprised age, sex, creatinine clearance (CCr), primary cancer site, Eastern Cooperative Oncology Group PS, body mass index (BMI), hemoglobin level, neutrophil‐to‐lymphocyte ratio (NLR), and site of metastasis at initiation of first‐line treatment. CCr was calculated using the Cockcroft–Gault formula as follows: CCr = ([{140 − age} × weight]/[72 × serum creatinine]) × 0.85 (if female). BMI was calculated as follows: BMI = weight/height^2^ (kg/m^2^).

### Statistical analysis

All statistical analyses were performed using JMP® Pro, Version 15.1.0 (SAS Institute Inc., Cary, NC, USA). Overall survival (OS) was defined as the time from the initiation of first‐line systemic treatment, excluding perioperative chemotherapy, or the date of the diagnosis in patients without treatment, to death from any cause. OS was estimated using Kaplan–Meier analysis with the log‐rank test. Multivariate logistic regression analysis was performed to identify the risk factors in patients who did not reach third‐line treatment. The cutoffs for hemoglobin and NLR were in accordance with those in a previous report.^9^ All tests were two‐sided, and *p* < 0.05 was considered statistically significant.

## RESULTS

### Details of sequential treatment in the entire cohort

**FIGURE 1:**
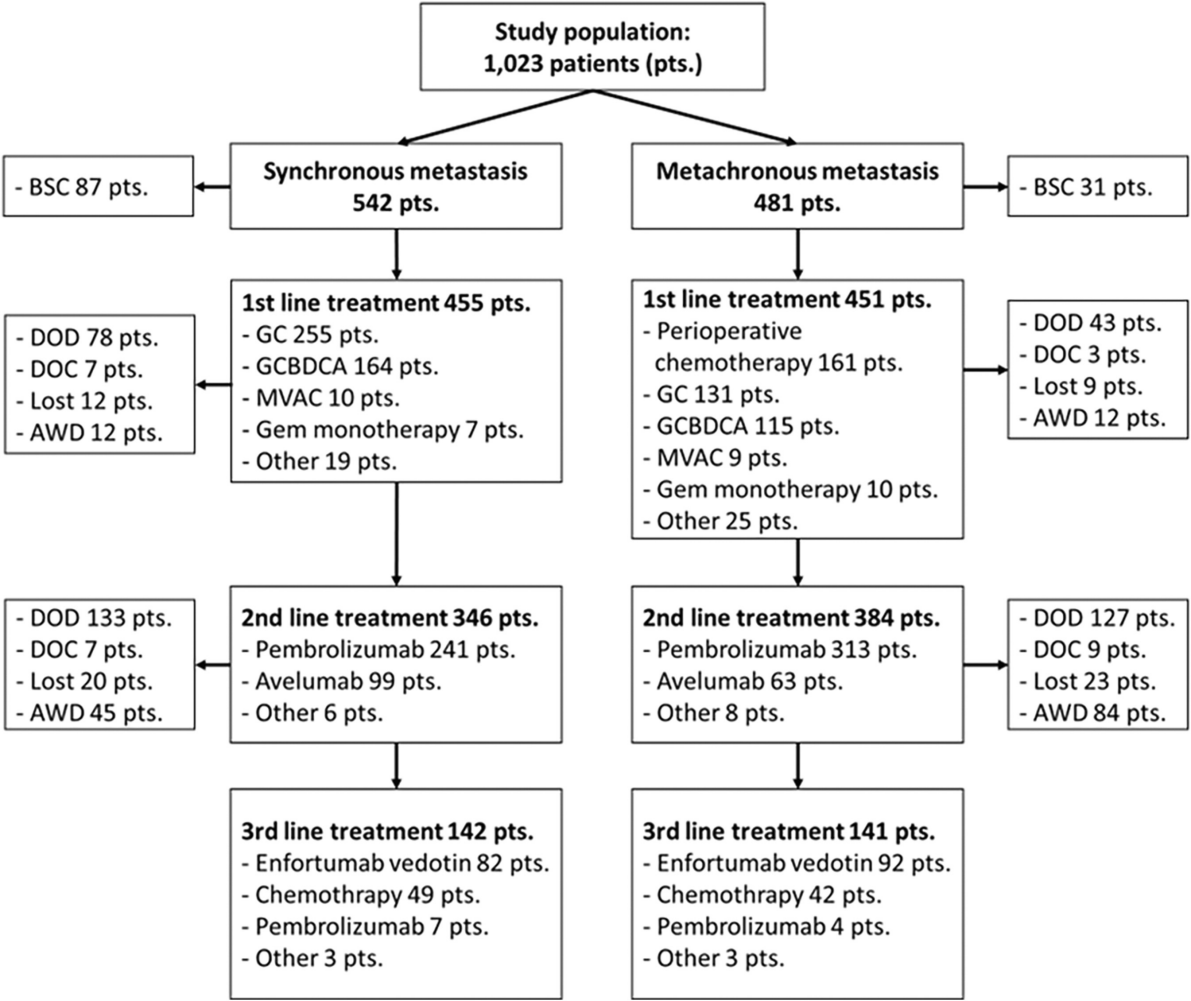
Flowchart of the patients included in this study. AWD, alive with disease; DOC, dead of other causes; DOD, dead of disease; GC, gemcitabine and cisplatin; GCBDCA, gemcitabine and carboplatin; MVAC, methotrexate, vinblastine, doxorubicin and cisplatin; Pts, patients.

**FIGURE 2:**
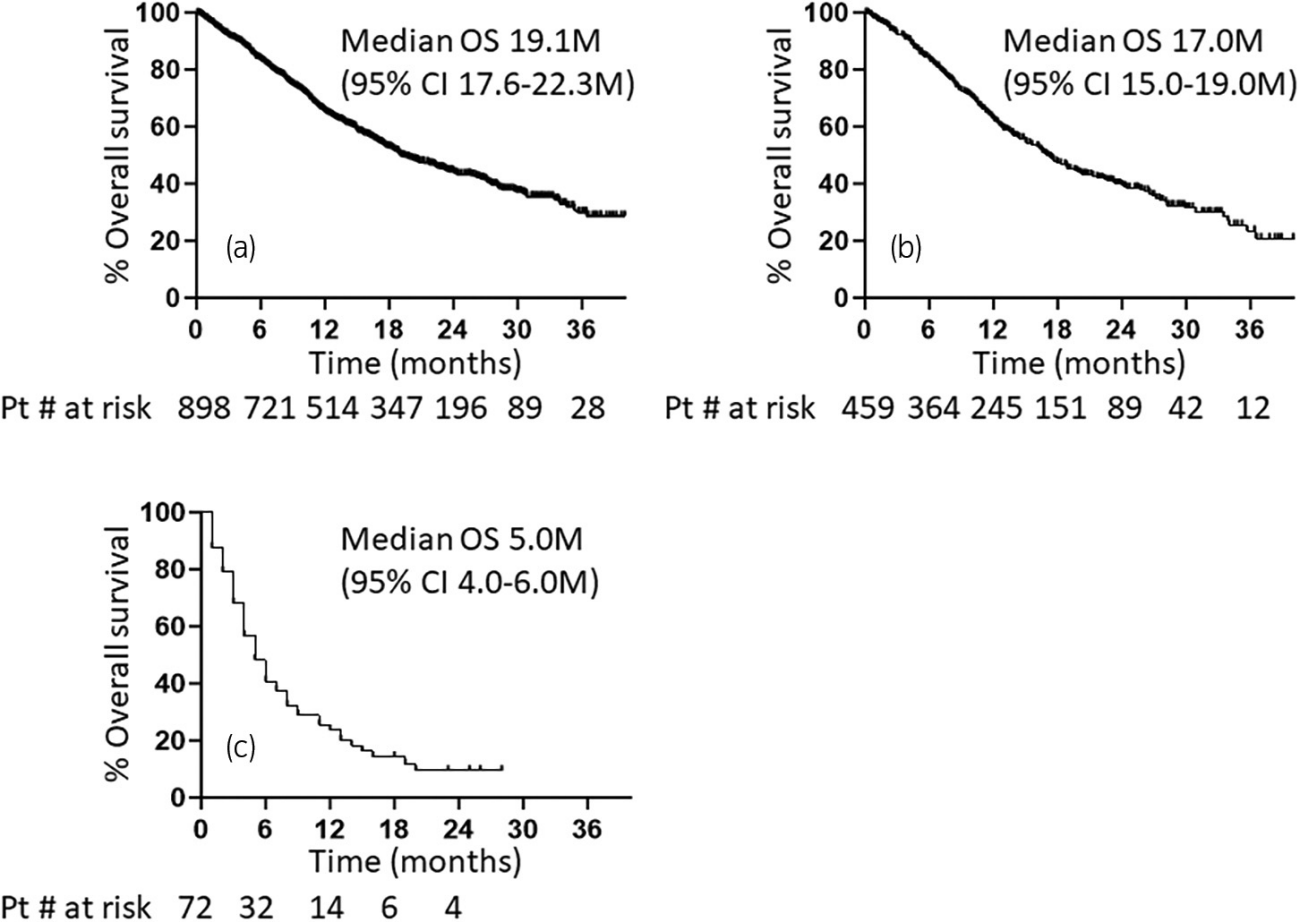
Kaplan–Meier plots displaying overall survival (OS) from initiation of systemic treatment for 898 patients in the entire cohort (a) and 459 patients with synchronous metastasis (b). (c) Kaplan–Meier plots showing OS from the diagnosis of metastatic urothelial carcinoma for 72 patients without systemic treatment. CI, confidence interval; M, months; Pt, patient.

The entire cohort of 1023 la/m UC patients comprised 542 patients in the synchronous metastasis group with unresectable or metastatic UC at diagnosis and 481 in the metachronous group with a history of primary tumor resection (Figure 1). In 118 of these cases, best supportive care (BSC) was selected on the basis of age, comorbidities, and patient preference; 905 received systemic treatment. At the median follow‐up of 28.5 months, the median OS from the initiation of first‐line treatment for patients with systemic treatment was 19.1 months (95% confidence interval [CI]: 17.6–22.3; Figure 2a). The median OS for patients in the synchronous metastasis group was 17.0 months (95% CI: 15.0–19.0; Figure 2b). Of the 118 patients who were selected for BSC, data on the survival outcome were available in 72 (61%). The median overall survival of the 72 patients was only 5.0 months (95% CI: 4.0–6.0; Figure 2c).

**FIGURE 3:**
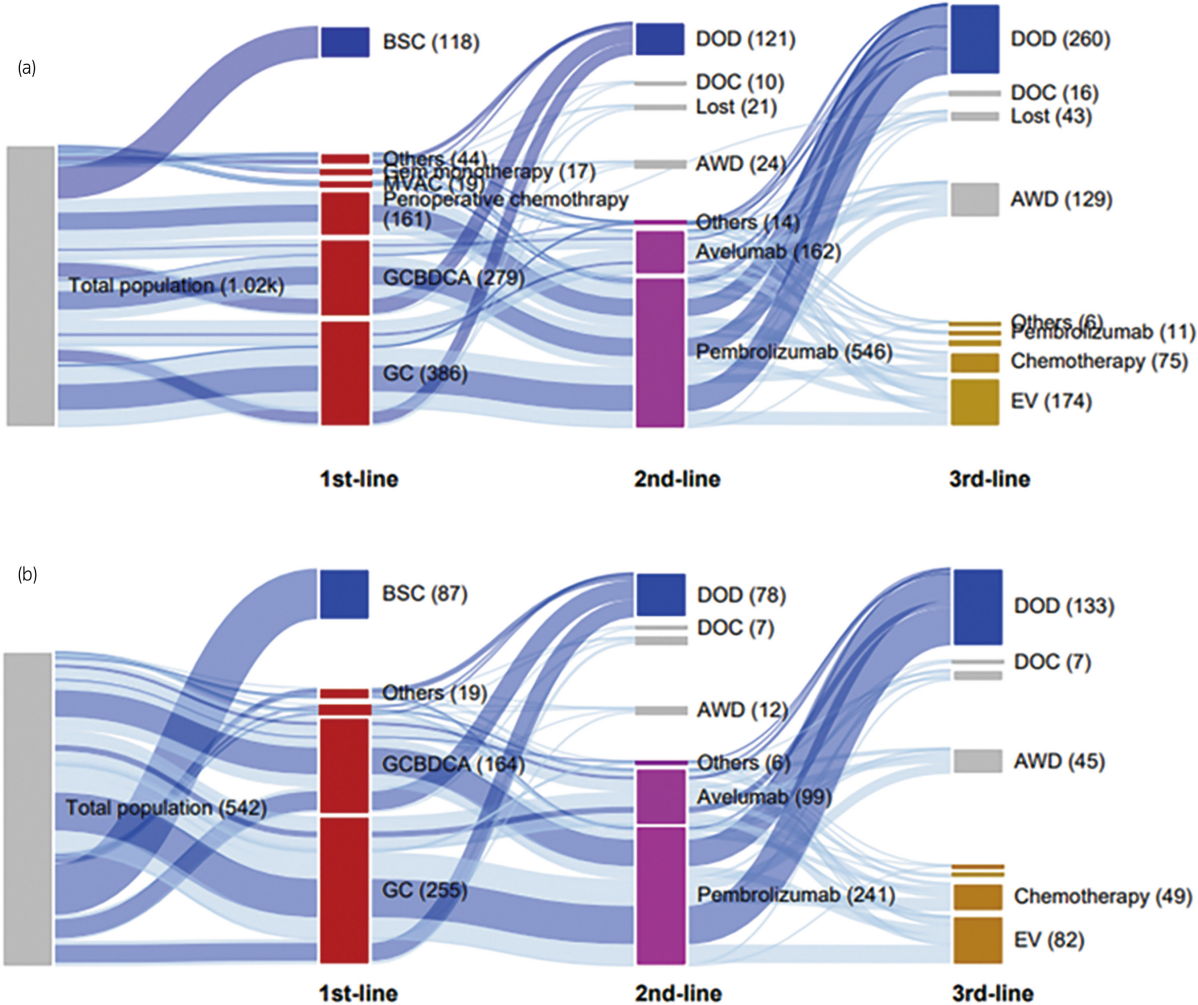
Sankey diagram of treatment choice for the 1023 patients in the entire cohort (a) and 542 patients with synchronous metastasis (b). AWD, alive with disease; BSC, best supportive care; DOC, dead of other causes; DOD, dead of disease; EV, enfortumab vedotin; GC, gemcitabine and cisplatin; GCBDCA, gemcitabine and carboplatin; MVAC, methotrexate, vinblastine, doxorubicin, and cisplatin.

Among the 905 patients with systemic treatment, 730 (81%) received second‐line treatment, and 283 (31%) proceeded to third‐line treatment (Figures 1 and 3a). Notably, 471 (52%) patients had their treatment terminated before the opportunity for third‐line treatment. To address differences in the patients' background data at the start of first‐line chemotherapy, we exclusively analyzed the data for the synchronous metastasis group. Among the 455 patients in this subgroup, 346 (76.0%) received second‐line treatment, while 142 (31.2%) advanced to third‐line treatment (Figures 1 and 3b). This analysis also revealed that 257 (56%) patients discontinued therapy before third‐line treatment. Consistent with these data, among patients who died of disease after January 2022, when EV became available, only 40 of 80 (50.0%) patients in the metachronous group and 54 of 106 (50.9%) patients in the synchronous group had received EV.

### Characteristics of patients who could not receive third‐line treatment in the synchronous metastasis group

The patients' baseline characteristics at the initiation of first‐line treatment in the synchronous group are summarized in Table 1. Of the 379 patients without missing data, 196 (51.7%) had died before receiving third‐line treatment. Multivariate analysis of the risk factors associated with patients not receiving third‐line treatment identified low PS (≥1), low BMI (<21 kg/m^2^), and high NLR (≥3) as significant risk factors for failure to reach third‐line treatment (Table 2).

**TABLE 1:** Patients' characteristics.

	*n* = 379
Age, years, median (range)	73 (35–91)
Age, *N* (%)	
≥75	162 (42.8)
<75	217 (57.2)
Sex, *N* (%)	
Male	270 (71.2)
Female	109 (28.8)
Creatinine clearance, *N* (%)	
≥50	122 (32.2)
<50	257 (67.8)
Primary site, *N* (%)	
Bladder/urethra	196 (51.7)
Upper tract	183 (48.2)
ECOG PS, *N* (%)	
0	199 (52.5)
1	139 (36.7)
≥2	41 (10.8)
BMI, median (range)	22.5 (14.3–32)
BMI, *N* (%)	
<21 kg/m^2^	128 (33.8)
21 < BMI ≤ 25 kg/m^2^	167 (44.1)
25 kg/m^2^≤	84 (22.1)
Metastatic site, *N* (%)	
Liver	48 (12.7)
Lung	107 (28.2)
Bone	79 (20.8)
Hb, *N* (%)	
≥11 g/dL	242 (63.9)
<11 g/dL	137 (36.1)
NLR, *N* (%)	
≥3	260 (68.6)
<3	119 (31.4)

Abbreviations: BMI, body mass index; BSC, best supportive care; ECOG PS, Eastern Cooperative Oncology Group performance status; Hb, Hemoglobin; LN, lymph node; NLR, neutrophil‐to‐lymphocyte ratio.

**TABLE 2:** Multivariate analysis of the factors associated with not receiving third‐line treatment.

	Odds	Lower 95%	Higher 95%	*p*‐Value
Age				
<75	Ref.			
≥75	1.5937	0.961	2.643	0.0709
Sex				
Male	Ref.			
Female	0.6849	0.4142	1.1324	0.1402
CCr				
≥50	Ref.			
<50	1.0753	0.6111	1.8922	0.801
Primary site				
Bladder/urethra	Ref.			
Upper tract	1.1757	0.7417	1.8635	0.4909
ECOG PS				
0	Ref.			
1	2.0012	1.2245	3.2707	0.0056
≥ 2	3.1052	1.3684	7.0463	0.0067
BMI				
21 ≤ BMI < 25	Ref.			
< 21	2.0221	1.1963	3.4178	0.0086
25≤	1.0517	0.5759	1.9205	0.8696
Lung metastasis				
No	Ref.			
Yes	1.2081	0.7252	2.0125	0.4677
Liver metastasis				
No	Ref.			
Yes	1.6646	0.8122	3.4115	0.1639
Bone metastasis				
No	Ref.			
Yes	1.1708	0.6693	2.0479	0.5803
Hb				
≥11 g/dL	Ref.			
<11 g/dL	1.3827	0.8495	2.2506	0.1922
NLR				
<3	Ref.			
≥3	1.9776	1.2024	3.2527	0.0072

Abbreviations: BMI, Body mass index; CCr, Creatinine clearance; ECOG PS, Eastern Cooperative Oncology Group performance status; Hb, Hemoglobin; NLR, neutrophil‐to‐lymphocyte ratio.

**FIGURE 4:**
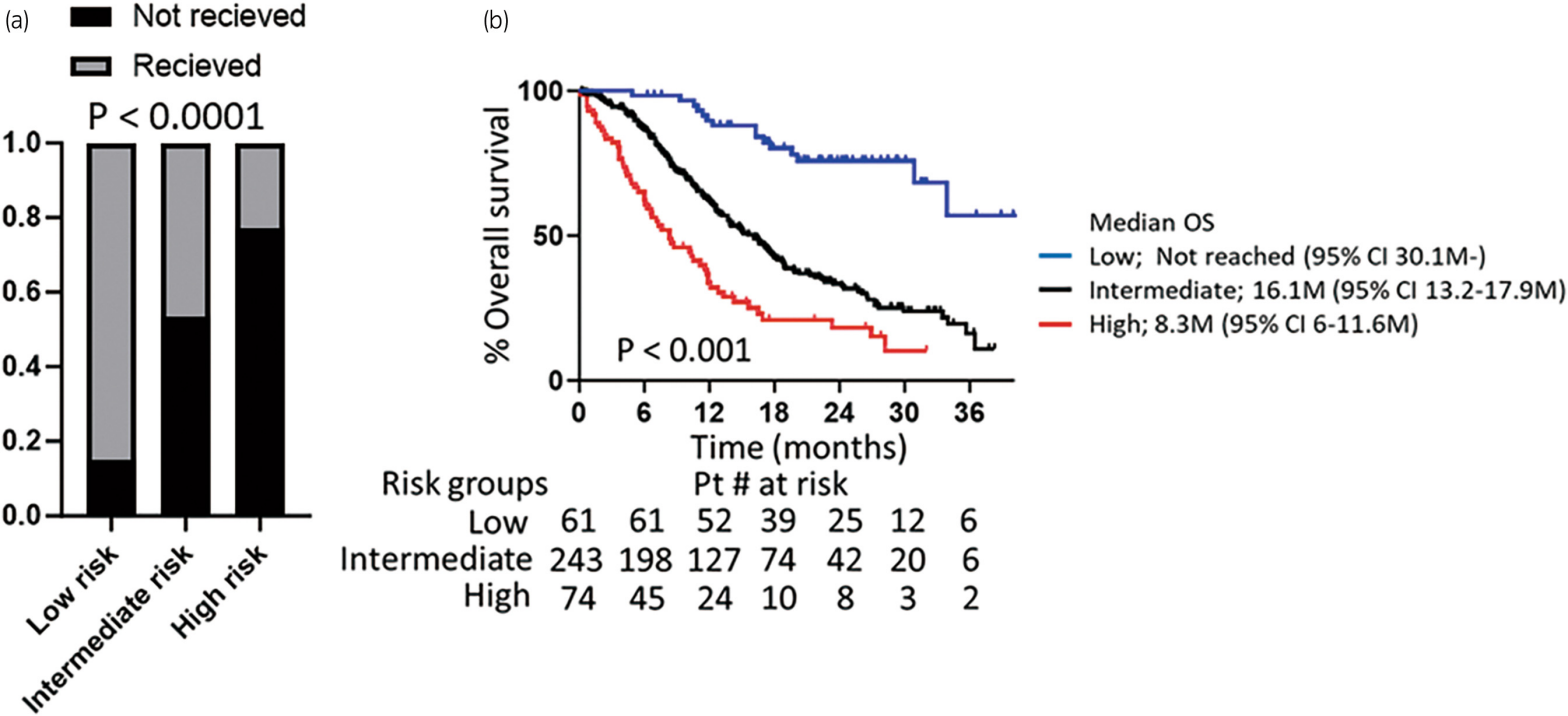
Risk classification on the basis of NLR, BMI, and PS correlates with the proportion of patients who can receive up to third‐line treatment (a), and OS (b). BMI, body mass index; M, months; NLR, neutrophil‐to‐lymphocyte ratio; OS, overall survival; PS, performance status; Pt, patient.

We assigned the following scores to the three dichotomous or trichotomous variables and incorporated them into predictive models as follows: NLR ≥3 (1) or <3 (0); BMI < 21 kg/m^2^ (1) or ≥21 kg/m^2^ (0); and Eastern Cooperative Oncology Group PS ≥2 (2), 1 (1), or 0 (0). In the three‐factor model, each patient was assigned a total risk score ranging from 0 to 4, and we divided the patients into three groups, namely low‐risk (score = 0, *n* = 61), intermediate‐risk (score = 1–2, *n* = 244), and high‐risk (score = 3–4, *n* = 74) groups. The results showed wide separations in the proportion of patients who could not receive third‐line treatment between the groups (*p* < 0.0001, chi‐square test; Figure 4a). The risk classification of the three‐factor model also yielded wide separations of the Kaplan–Meier curves for OS between the groups (*p* < 0.001, log‐rank test; Figure 4b).

## DISCUSSION

Using real‐world data, the present study clarified the median OS for la/mUC patients in Japan, showing that more than half of the patients failed to reach third‐line treatment despite participation in a universal healthcare system.

The sequential treatment of la/mUC has changed dramatically over the past decade with the introduction of several new agents. Clinical trial data supporting drug approval showed that pembrolizumab prolonged survival by approximately 3 months as second‐line therapy after failure of platinum‐based chemotherapy,^1^ and EV prolonged prognosis by approximately 4 months as third‐line treatment after failure of platinum‐based chemotherapy and immune checkpoint inhibitors.^3^ Avelumab has also been shown to prolong prognosis by approximately 7 months as maintenance therapy after platinum‐based chemotherapy.^2^ However, the 17 months of OS from the start of first‐line treatment that we identified in this study is not as long as one would expect, compared with the 14–15 months' survival from the era when chemotherapy was the only treatment available.^10^ Indeed, while OS shown in our previous report for those who received subsequent chemotherapy after second‐line pembrolizumab in pre‐EV era was 13.3 (95% CI 10.5–19.8) months,^11^ OS shown in the present study for those who received subsequent EV in the post‐EV era was 12 months (95% CI 10.8–). Of course, it must be mentioned that it is currently difficult to compare these two cohorts due to several crucial biases that are difficult to eliminate using the available data including variations in treatment processes leading up to the initiation of third‐line treatment. Nonetheless, it doesn't seem like there's been any dramatic improvement in patient survival between pre‐ and post‐EV eras from the currently available data so far. This appears to reflect the fact that urothelial carcinoma progresses rapidly, and many patients die before benefiting from second‐ or third‐line treatment. In fact, our data showed that approximately 80% of the patients were eligible to receive immune checkpoint inhibitors as second‐line therapy, but less than 50% survived to third‐line therapy. This proportion is better than that of the previous report from the United States^8^ in which first‐line, second‐line and third‐line systemic anticancer treatment was implemented in only 48, 17, and 6% of patients who were diagnosed la/m UC, respectively. Although this is primarily attributed to the lack of availability of evidence‐based treatment, other issues on social healthcare systems including economic reasons and limited access to medical care were also cited as contributing factors. The present study on patients treated in Japan reveals that medical causes make it difficult to carry out sequential treatment for la/mUC to the end, even when the factors of economic reasons and limited access to medical care are excluded.

We identified poor PS, high NLR, and low BMI as risk factors for failure to reach third‐line treatment. PS is a prognostic factor both at the start of first‐line chemotherapy^12^ and at the start of pembrolizumab treatment,^9^ while NLR has recently received attention as a marker of systemic inflammatory status and is a prognostic factor for chemotherapy and pembrolizumab treatment.^13^, ^14^ Therefore, it is reasonable that these risk factors were important in our study, in the execution of sequential treatment. Interestingly, low BMI was also identified as an independent risk factor, whereas high BMI or obesity was not. Low BMI correlates with a patient's frailty status and is included in functional assessment tools for the elderly, such as the G8, which has recently received much attention.^15^, ^16^ This finding suggests that frailty status remains a negative factor for treatment adherence. Notably, BMI and PS were identified as risk factors rather than liver metastasis, which is often reported as a prognostic factor for chemotherapy and pembrolizumab.^14^, ^17^ This finding appears to indicate that patient‐related factors have a stronger impact on the execution of sequential therapy compared with tumor‐related factors.

To improve treatment tolerability and prolong prognosis, nutritional interventions may be useful. However, if the cause of low BMI is inflammatory cytokine‐mediated metabolic changes due to cancer, as suggested by high NLR values, the effect of nutritional interventions may be limited. Our proposed risk classification, which combines NLR, PS, and BMI, can easily estimate the likelihood of reaching third‐line treatment, and correlates with patient prognosis. For high‐risk patients, sequential treatment should be initiated in addition to early preparation for the end stages of cancer. These preparations may include home care and hospice arrangements, which should be considered at the same time as sequential treatment, with consideration of nutritional supportive care.

There is currently no strong evidence for the sequential order of treatment. A major barrier is the lack of well‐established biomarkers to predict the efficacy of chemotherapy and immune checkpoint inhibitors prior to administration. The order of sequential therapy and novel combination therapies are currently being tested in several clinical trials, and results are anticipated. In particular, it would be interesting to know whether intermediate‐ or high‐risk patients in this study would have benefitted more from early treatment with immune checkpoint inhibitors or EV. In this respect, the Japanese dataset, which, unlike that in the USA, has less variation in sequential treatment because immune checkpoint inhibitors are not available for first‐line treatment, is highly valuable for comparisons with other cohorts.

There are several limitations to this study in addition to its retrospective design. First, the number of patients who were selected for BSC without systemic treatment may have been underestimated because the eligible patients were extracted retrospectively from medical record information. Second, the timing and details of sequential treatment changes are based on the judgment of each treating institution and are not standardized. Third, we did not validate our proposed risk classification in an external cohort; therefore, future validation is needed. Fourth, as avelumab and EV became available during the enrollment period of this cohort, the sequential treatment pattern differs between patients who started treatment early in the enrollment period and those started late. Although it is desirable to evaluate frailty using a more specific index such as the frailty index, this study could only use PS and BMI as substitutes. Nonetheless, this study provides important information for sequential treatment of la/mUC in Japan and will serve as control data for future revisions of sequential treatment.

## AUTHOR CONTRIBUTIONS

**Yuki Kita:** Conceptualization; methodology; data curation; formal analysis; writing—original draft; investigation; software; visualization. **Hikari Otsuka:** Data curation; writing—review & editing; investigation. **Katsuhiro Ito:** Writing—review & editing; software. **Takuto Hara:** Investigation; writing—review & editing. **Soichiro Shimura:** Investigation; writing—review & editing. **Takashi Kawahara:** Investigation; writing—review & editing. **Minoru Kato:** Investigation; writing—review & editing. **Sojun Kanamaru:** Investigation; writing—review & editing. **Koji Inoue:** Investigation; writing—review & editing. **Hiroki Ito:** Investigation; writing—review & editing. **Atsushi Igarashi:** Investigation; writing—review & editing. **Tomokazu Sazuka:** Investigation; writing—review & editing. **Dai Takamatsu:** Investigation; writing—review & editing. **Kohei Hashimoto:** Investigation; writing—review & editing. **Takashige Abe:** Investigation; writing—review & editing. **Sei Naito:** Investigation; writing—review & editing. **Yoshiyuki Matsui:** Investigation; writing—review & editing. **Hiroyuki Nishiyama:** Writing—review & editing; supervision. **Hiroshi Kitamura:** Supervision; writing—review & editing. **Takashi Kobayashi:** Writing—review & editing; conceptualization; supervision; methodology.

## CONFLICT OF INTEREST STATEMENT

The authors declare no conflict of interest.

## APPROVAL OF THE RESEARCH PROTOCOL BY AN INSTITUTIONAL REVIEWER BOARD

The protocol for this research project has been approved by the Institutional Review Board of Kyoto University Graduate School of Medicine (approval number R3245) and the local Institutional Review Boards at each participating institute and it confirms to the provisions of the Declaration of Helsinki.

## INFORMED CONSENT

Not applicable.

## REGISTRY AND THE REGISTRATION NO. OF THE STUDY/TRIAL

Not applicable.

## ANIMAL STUDIES

Not applicable.

## Appendix

Study institutes: Osaka Metropolitan University, Akita University, Hirosaki University, National Cancer Center Hospital, Hamamatsu University School of Medicine, Yamagata University Faculty of Medicine, Kyoto University, University of Tsukuba, Nara Medical University, Shizuoka General Hospital, University of the Ryukyus, Iwate Medical University, Oita University, Hiroshima University, University of Yamanashi, Japanese Red Cross Wakayama Medical Center, Kobe City Nishi‐Kobe Medical Center, Japanese Red Cross Osaka Hospital, Nagoya University, Harasanshin Hospital, Hokkaido University, Japanese Red Cross Otsu Hospital, Kagoshima University, Kyushu University, Shikoku Cancer Center, Tenri Hospital, Hakodate Goryoukaku Hospital, Kitasato University, Kyoto Katsura Hospital, National Hospital Organization Kyoto Medical Center, Kumamoto University, National Hospital Organization Himeji Medical Center, Tazuke Kofukai Medical Research Institute, Kitano Hospital, Toyooka Hospital, University of Miyazaki, Hitachi General Hospital, Mie University, Ibaraki Prefectural Central Hospital, Ijinkai Takeda General Hospital, University of Toyama, Otsu City Hospital, Sapporo Medical University, Kansai Electric Power Hospital, Hyogo College of Medicine, Hirakata Kohsai Hospital, Jikei University, Tokushima University, Aichi Medical University, Shiga General Hospital, Juntendo University, Chiba University, Kobe City Medical Center General Hospital, Yokohama City University, Kobe University, and Kurashiki Central Hospital.
